# Supplementary figures and images for: Comparison of machine learning methods in forecasting and characterizing the birch and grass pollen season
Source: PLoS One. 2026 Feb 18;21(2):e0332093. doi: 10.1371/journal.pone.0332093 (PMC12915917; doi:10.1371/journal.pone.0332093)

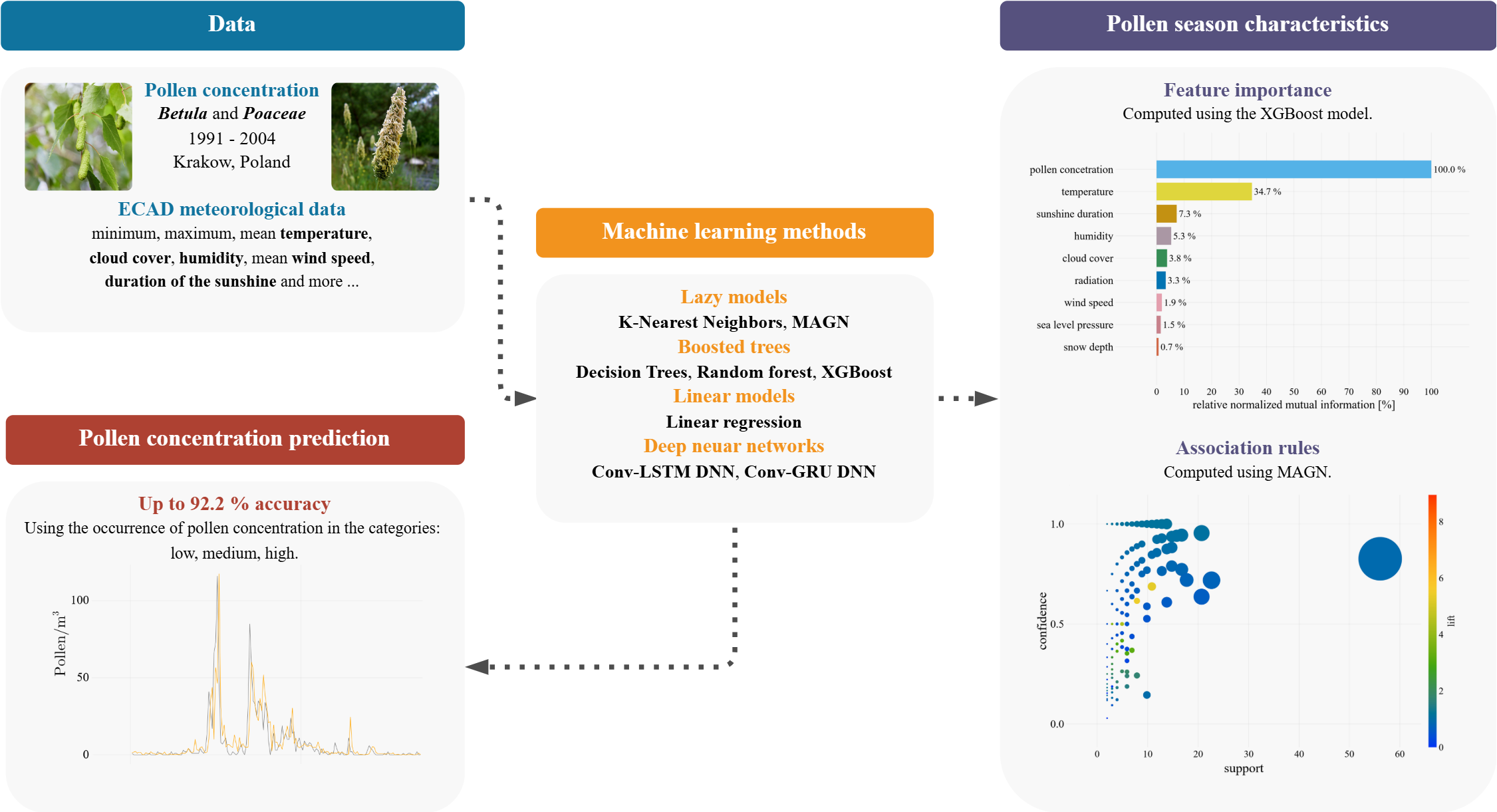

Supplement: S1 Fig — (TIF) [file pone.0332093.s001.tif]
