# Supplementary material for: Comparison of machine learning methods in forecasting and characterizing the birch and grass pollen season
Source: PLoS One. 2026 Feb 18;21(2):e0332093. doi: 10.1371/journal.pone.0332093 (PMC12915917; doi:10.1371/journal.pone.0332093)
Supplement: S2 File — (DOCX) [file pone.0332093.s002.docx]

**Machine learning methods**

- MAGN
  - fuzzify_input_data: true
  - winners_limit_ratio_classification: 0.78 - 0.83
  - winners_weight_exponent: 1
  - intersensory_activation_threshold: 0.1
  - intersensory_activation_exponent: 10
  - similarity_threshold: 0.9
  - weighting_strategy: "AlwaysOne"
- Decision Trees
  - max_depth: -1
  - min_samples_leaf: 1
  - min_samples_split: 2
  - min_purity_increase: 0.0
  - n_subfeatures: 0
  - post_prune: false
  - merge_purity_threshold: 1.0
  - display_depth: 5
  - feature_importance: :impurity
  - rng: TaskLocalRNG
- Random Forest
  - n_estimators: 100
  - criterion: "gini"
  - max_depth: 25
  - min_samples_split: 2
  - min_samples_leaf: 1
  - min_weight_fraction_leaf: 0.0
  - max_features: "sqrt"
  - max_leaf_nodes: nothing
  - min_impurity_decrease: 0.0
  - bootstrap: true
  - oob_score: false
  - n_jobs: nothing
  - random_state: nothing
  - verbose: 0
  - warm_start: false
  - class_weight: nothing
  - ccp_alpha: 0.0
  - max_samples: nothing
  - monotonic_cst: nothing
- XGBoost
  - test: 1
  - num_round: 100
  - booster: "gbtree"
  - disable_default_eval_metric: 0
  - eta: 0.3
  - num_parallel_tree: 1
  - gamma: 0.0
  - max_depth: 25
  - min_child_weight: 1.0
  - max_delta_step: 0.0
  - subsample: 1.0
  - colsample_bytree: 1.0
  - colsample_bylevel: 1.0
  - colsample_bynode: 1.0
  - lambda: 1.0
  - alpha: 0.0
  - tree_method: "auto"
  - sketch_eps: 0.03
  - scale_pos_weight: 1.0
  - updater: nothing
  - refresh_leaf: 1
  - process_type: "default"
  - grow_policy: "depthwise"
  - max_leaves: 0
  - max_bin: 256
  - predictor: "cpu_predictor"
  - sample_type: "uniform"
  - normalize_type: "tree"
  - rate_drop: 0.0
  - one_drop: 0
  - skip_drop: 0.0
  - feature_selector: "cyclic"
  - top_k: 0
  - tweedie_variance_power: 1.5
  - objective: "automatic"
  - base_score: 0.5
  - early_stopping_rounds: 0
  - watchlist: nothing
  - nthread: 1
  - importance_type: "gain"
  - seed: nothing
  - validate_parameters: false
- KNN
  - K: 5
  - algorithm: :kdtree
  - metric: Euclidean
  - leafsize: 10
  - reorder: true
  - weights: Uniform
- Linear Regression:
  - fit_intercept: true
  - solver: nothing
- Conv-LSTM DNN
  - optimiser = Adam(5.0e-5, (0.9, 0.999), 1.0e-8)
  - loss = Flux.Losses.mse
  - epochs = 100
  - batch_size = 1
  - lambda = 0.0
  - alpha = 0.0
  - rng = TaskLocalRNG()
  - optimiser_changes_trigger_retraining = false
- Conv-GRU DNN
  - optimiser = Adam(5.0e-5, (0.9, 0.999), 1.0e-8)
  - loss = Flux.Losses.mse
  - epochs = 100
  - batch_size = 1
  - lambda = 0.0
  - alpha = 0.0
  - rng = TaskLocalRNG()
  - optimiser_changes_trigger_retraining = false
