# Supplementary material for: Comparison of machine learning methods in forecasting and characterizing the birch and grass pollen season
Source: PLoS One. 2026 Feb 18;21(2):e0332093. doi: 10.1371/journal.pone.0332093 (PMC12915917; doi:10.1371/journal.pone.0332093)
Supplement: Attachment — Submitted filename: Response to Reviewers.pdf [file pone.0332093.s003.pdf]

Response to Reviewers

Manuscript title: *Comparison of machine learning methods in forecasting and characterizing the birch and grasses pollen season*
Manuscript ID: PONE-D-25-12434
Journal: *PLOS ONE*

Dear Editor and Reviewers,

We would like to thank the Academic Editor and both reviewers for their constructive feedback on our manuscript. We appreciate the time and effort invested in the review process, and we have revised the manuscript accordingly to address all points raised.

Below, we provide a detailed point-by-point response to the reviewers' and editor's comments. All changes have been incorporated into the revised version of the manuscript, which is submitted along with a tracked-changes file for reference.

General Editorial Requirements

1. **Please ensure that your manuscript meets PLOS ONE's style requirements, including those for file naming. The PLOS ONE style templates can be found at https://journals.plos.org/plosone/s/file?id=wjVg/PLOSOne_formatting_sample_main_body.pdf and https://journals.plos.org/plosone/s/file?id=ba62/PLOSOne_formatting_sample_title_authors_affiliations.pdf**

   We have revised the manuscript to comply with *PLOS ONE* style and formatting guidelines. In particular, the Supporting information section has been moved to the end of the manuscript and the description of the corresponding author has been corrected.

2. **Please note that PLOS ONE has specific guidelines on code sharing for submissions in which author-generated code underpins the findings in the manuscript. In these cases, we expect all author-generated code to be made available without restrictions upon publication of the work. Please review our guidelines at https://journals.plos.org/plosone/s/materials-and-software-sharing#loc-sharing-code and ensure that your code is shared in a way that follows best practice and facilitates reproducibility and reuse.**

   We have deposited all necessary author-generated code to GitHub (https://github.com/bionetlabs/PollenForecasting) to ensure unrestricted public access. Appropriate information has been placed in the Materials and methods section.

3. **Thank you for uploading your study's underlying data set. Unfortunately, the repository you have noted in your Data Availability statement does not qualify as an acceptable data repository according to PLOS's standards.**

At this time, please upload the minimal data set necessary to replicate your study's findings to a stable, public repository (such as figshare or Dryad) and provide us with the relevant URLs, DOIs, or accession numbers that may be used to access these data. For a list of recommended repositories and additional information on PLOS standards for data deposition, please see https://journals.plos.org/plosone/s/recommended-repositories.

The whole material, including row data, pollen data, meteorological data and all the obtained results were stored as project documentation by the Jagiellonian University Medical College.
The meteorological data were collected from the European Climate Assessment and Dataset website [https://www.ecad.eu] as blended data in ASCII file format. All observations were obtained from the Krakow-Balice station. The direct link to the data Policy is as follows: https://knmi-ecad-assets-prd.s3.amazonaws.com/documents/ECAD_datapolicy.pdf.
We acknowledge the data providers in the ECA&D project.
Klein Tank, A.M.G. and Coauthors, 2002. Daily dataset of 20th-century surface air temperature and precipitation series for the European Climate Assessment. Int. J. of Climatol., 22, 1441-1453.
Moreover, to provide a minimal data set necessary to replicate our study, two csv files with *Betula* pollen and two files with *Poaceae* pollen data obtained in 2022 and 2023, and the meteorological data of ten selected factors were uploaded to the Open Science Framework with DOI number: DOI 10.17605/OSF.IO/9YZCF and the direct link: https://osf.io/9yzcf/?view_only=d4ccc99d460c46bfa775d72ba6ba3a1b.
Appropriate information has been placed in the Materials and methods section.

4. **While revising your submission, please upload your figure files to the Preflight Analysis and Conversion Engine (PACE) digital diagnostic tool, https://pacev2.apexcovantage.com/. PACE helps ensure that figures meet PLOS requirements. To use PACE, you must first register as a user. Registration is free. Then, login and navigate to the UPLOAD tab, where you will find detailed instructions on how to use the tool. If you encounter any issues or have any questions when using PACE, please email PLOS at figures@plos.org. Please note that Supporting Information files do not need this step.**

We have uploaded all main-text figure files to the PACE diagnostic tool and confirmed that they now comply with PLOS figure requirements.

Academic Editor Comments

1. **The background and objectives need to be better described.**

We have significantly revised the Introduction to better describe the background, the motivations for applying machine learning to aerobiological forecasting, and the gap

in current literature. Specific objectives of the study are now clearly defined at the end of the Materials and Methods section.

Reviewer #1 Comments

1. **While meteorological data is included, there is no mention of potential measurement errors or quality variations, which might affect the model's precision. The methodology section could benefit from greater clarity and detail. For example, the description of data preprocessing techniques and modeling strategies could be expanded to include more information on the specific parameters used. In particular, the Authors could to clarify: - The distance between the weather station and the pollen sampling point (over 11 km) could create discrepancies between collected data and actual conditions relevant to the study area.**

Thank you for pointed out the problem related to the distance between the meteorological and aerobiological stations. We are aware, that the microclimate conditions can differ between both stations, but the main reasons of taking into account the meteorological data from Balice station was that these data are commonly available. It is important not only during the models preparation, but also in the nearest future, when the models will be implemented into the aerobiological data base to predict the pollen concentrations up to date.
For our best knowledge, based on the reports on the meteorological data in both stations (Balice and Kraków city center) the differences between them are relatively low. According to Matuszko et al. (2015) and Matuszko and Piotrowicz (2015), in 2001-2010 annual temperature in Kraków center was higher by 0.7 °C in comparison to Balice station, whereby the differences were observed mainly in winter, while the pollen seasons are finished. The other data, like sunshine, cloud cover, annual relative humidity (77% vs 78%, Kraków and Balice, respectively), were slightly different.
We now discuss the potential impact of measurement errors and spatial discrepancy between the meteorological station and pollen trap in the *Materials and Methods* under *Data* subsection. In addition, we have added detailed descriptions of the experimental design and validation procedures to the Materials and Methods section.

Matuszko D., Piotrowicz K., Kowanetz L., 2015, Klimat, [w:] Środowisko przyrodnicze Krakowa. Zasoby, Ochrona, Kształtowanie, Baścik M., Degórska B. (red.), Instytut Geografii i Gospodarki Przestrzennej UJ, Kraków, 81-108.
Matuszko D., Piotrowicz K., 2015, Cechy klimatu miasta a klimat Krakowa, [w:] Miasto w badaniach geografów, Trzepacz P., Więcław-Michniewska J., Brzosko-Sermak A., Kołoś A. (red.), T. 1, Instytut Geografii i Gospodarki Przestrzennej UJ., Kraków, 221-241.

2. **The model relies solely on data from Krakow over a span of 34 years, limiting its applicability to regions with different climatic conditions. It could be useful to comment about that in section weakness or discussion for example.**

This limitation is acknowledged and discussed in the *Discussion* section.

3. **Machine Learning Models: The "default settings" approach for model parameters may restrict performance. Exploring diverse hyperparameter settings would have been beneficial for optimization. In particular, the manuscript does not discuss in detail the impact of data quality (e.g., measurement errors) or the necessity of hyperparameter optimization, which could improve the models' performance.**

We appreciate the Reviewer's 5 insightful comment. Indeed, exploring the full hyperparameter space could further improve model performance. However, given the large number of models and variants (forecast horizons, taxa), we adopted a standardized pipeline with default parameters to ensure consistency and comparability across all methods.
To address the reviewer's suggestion, we have now added a discussion in the manuscript highlighting this limitation and acknowledging that more exhaustive hyperparameter tuning could yield better results in some cases. Nonetheless, the models already achieve high performance (e.g., up to 92.2% accuracy), which we believe is sufficient to demonstrate their practical applicability in the context of daily pollen forecasting.
Moreover, regarding data quality, we ensured that the input dataset was thoroughly preprocessed following standardized aerobiological protocols, and we employed robust methods such as moving averages and mutual information analysis to mitigate the effect of noise. This has been clarified in the revised discussion section.

4. **Variable Interpretation: - The feature importance analysis appears detailed but could be expanded to include overlooked or underestimated influential variables**

We appreciate the reviewer's suggestion to further examine secondary predictors. Accordingly, we analyzed relative normalized mutual information for the lowest-ranking meteorological variables: humidity, sunshine duration, radiation, cloud cover, wind speed, sea level pressure, and snow depth. Although these factors fall below the primary drivers in overall rank, they nonetheless contribute meaningfully to model performance. A new paragraph in the Results section now summarizes these insights.

5. **Clinical Goal vs Results: Despite the clinical focus on predicting pollen concentrations for personalized treatment, the manuscript does not assess the practical impact of these forecasts on patient health outcomes. It could be useful to comment about that in section weakness or discussion for example.**

Thank you very much for drawing attention to the clinical aspect of our work. The practical implication of predicting pollen concentration is the possibility of intensifying antiallergic treatment and avoid activities outdoors at critical moments of pollen concentration to prevent the exacerbation of clinical symptoms in patients. The first stage of our work was the construction and evaluation of models, which is presented in this publication. In the further stages of the project, we plan to use the presented models to build a mobile application that will predict pollen concentrations and will be made available to patients. An appropriate note on this topic has been added to the Discussion section.

6. **Temporal Overlap: A clear explanation is missing regarding how previous pollen and weather data are synchronized for predictions. Potential temporal overlaps between variables may introduce bias into the models. It could be useful to comment about that in section weakness or discussion for example.**

   We thank the reviewer for highlighting the need to clarify how our input windows avoid using future information. In the revised manuscript, we have discussed the potential for autocorrelation bias in the Discussion.

7. **Influence of Meteorological Factors: In some parts of the manuscript, it is stated that temperature is the most important environmental factor influencing pollen concentration. However, in other sections, it is suggested that humidity and cloud cover may be of lesser importance. This contradiction could be resolved by providing a clearer and more consistent explanation.**

   We have revised the relevant paragraphs to present a more coherent interpretation. In particular, wed have expanded the description of Experiment 2 to clearly define its aims and analytical steps and we reworked the appropriate paragraphs in the Results section.

Reviewer #2 Comments

1. **The introduction is very reduced and exposed in very general lines. I think the authors could analyse a brief background of the models of machine learning used in aerobiological studies to predict pollen concentrations, main advantages in comparison to traditional statistical methods and accuracy achieved in the forecasting. Below several interesting and recent related studies using machine learning methods or combination of these methods which is now in practise. After this background, the discussion section should incorporate the main novelty and the most important findings of this study.**

   After correction, the Introduction includes a concise review of the use of machine learning in aerobiology, with emphasis on prior applications and comparative

performance. We have also enriched and emphasized the main novelty and the most important findings of this study in the Discussion section.

2. **The authors show how pollen time series and meteorological series follow a normal distribution which is strange in the aerobiological field due to the high frequency of zeros and low pollen concentrations during the year. Could you explain this? Anyway, normality assumptions would be only necessary in the case of linear models, are machine learning methods sensitive to the non-normal distribution of data? Discuss this aspect. Perhaps an advantage for using this type of forecasting methods.**

We thank the reviewer for this important observation. To clarify, we first excluded all calendar days outside the defined birch and grass pollen seasons. Specifically, those days before the pollen season began and after it ended, when pollen concentrations are zero. After this seasonal trimming, the remaining daily pollen and meteorological values deviate significantly from a normal distribution, as confirmed by Shapiro–Wilk test. We owe you an apology and a clarification regarding our discussion of data normality in the submitted manuscript. Due to a misunderstanding of the Shapiro–Wilk test output and the associated package documentation, we incorrectly reported that both the pollen concentration and meteorological datasets were normally distributed. In fact, the very low p-values ($p \ll 0.05$) from our Shapiro–Wilk tests demonstrate that these data strongly deviate from normality. We regret this error and have revised the manuscript to remove the incorrect statements about normality, instead applying and describing appropriate non-parametric analyses. Thank you for catching this mistake, and we appreciate your understanding as we correct our interpretation.
Furthermore, we agree that strict normality is only required for parametric inference in linear regression. In contrast, the remaining models used in our study are non-parametric and thus do not assume any particular distribution of inputs or residuals.

3. **Check the aerobiological terminology of the entire manuscript following standardized terminology in this scientific field (Galán et al., 2017).**

Thank you for this comment. The aerobiological terminology was adopted to the recommendations proposed by Galan et al. (2017).

4. **In my opinion, the experiment 2 is not well explained. Indicate better the objectives for each experiment followed.**

We appreciate this feedback and have expanded the description of Experiment 2 to clearly define its aims and analytical steps. Specifically, Experiment 2 was designed to (1) identify which features drive the predictions of our best-performing model, (2) quantify the strength of association between inputs and pollen outcomes via mutual information, and (3) uncover characteristic combinations of meteorological and

pollen variables using association rule mining algorithm based on MAGN. We have updated the manuscript to reflect these distinct objectives.
In addition, we have expanded the description of Experiment 1 to state its precise goals, data preparation steps, modeling tasks, and evaluation framework.

5. **The results in the current form is a simple description of figures and tables. In my opinion, the result sections should be a more elaborated presentation of the most important findings of the models.**

   We have substantially enhanced the Results for Experiment 1 by moving beyond simple recitation of tables and figures to provide interpretive insights into each model's performance, highlighting major trends and comparative strengths.
   The Results section for Experiment 2 has been updated to provide a more in-depth description of relative normalized mutual information, including the comparative rankings of each predictor, and the implications for understanding variable relationships.

6. **Only as a suggestion. It would be interesting to generate a comparative table based on the advantages and disadvantages of the different machine learning methods used in this work. It is a good and useful result for new similar studies**

   Thank you for this suggestion. In the Discussion section we have added Table 5 summarizing the advantages and disadvantages of the various ML models used, aiding interpretability for future studies.

7. **Conclusion section should remark the most important findings and the advances in the knowledge, but in the current form, part of the conclusions are general aspects already previously indicated.**

   The Conclusion section has been rewritten to emphasize the novelty and key findings of the work.

Reviewer #2 – Specific Comments

1. **Abstract: "pollens": The word "pollen" has both a singular and plural sense.**

   Corrected to "pollen".

2. **Abstract: "among others": What about the rest of meteorological parameters? Are not relevant?**

   We replaced this with a specific listing of the meteorological parameters used: temperature (mean, minimum and maximum), humidity, cloud cover, sunshine

duration, mean wind speed, mean sea level, global radiation and snow depth.

3. **Materials and methods (line 30): It is ambiguous. I guess this is the extension of the municipality area.**

We have clarified that these figures refer to the administrative area and population of the city of Krakow.

4. **Materials and methods (line 48): In "isuploaded" a space is required.**

Corrected.

5. **Materials and methods (Figure 1): Figure 1 is not informative due to the long time series. Perhaps, it would be more useful representing the daily average during the entire series adding lines for the first and third daily quartile to show the variability, or a similar graph.**

Replaced with a revised figure showing the median pollen count for each day of the year, together with the first (25th percentile) and third (75th percentile) quartiles, illustrating the typical seasonal peak and interannual variability.

6. **Materials and methods (Table 1): Replace "seeds/m3" by "pollen grains/m3"." and "Materials and methods (line 113-120): Replace "seeds/m3" by "pollen grains/m3".**

Changed to "Pollen/m^3" throughout the manuscript according to recommendations proposed by Galan et al. (2017).

7. **Materials and methods (line 79-80): What type of meteorological forecast are used to predict pollen in the future? This is relevant.**

To isolate the performance of our machine learning framework from the uncertainty of real-time forecasts, we did not use an operational weather-forecast model. Instead, in all experiments we simulated meteorological forecasts by using the corresponding historical observational data. That is, for a prediction horizon of n days and predefined time window w, we treated the recorded meteorological measurements on the target day and the preceding $n + w$ days as if they were forecast inputs. This approach ensures that any error in the pollen predictions stems from the modeling process itself, rather than from errors in external forecast data.

8. **Materials and methods (line 113-120): What is the meaning of the percentages of each pollen threshold? Is it related to the calculation of the threshold explained in lines 108-110.**

Each of the pollen threshold is related to the intensity of symptoms in sensited persons. They were calculated as presented in lines 108-110 (followed by Myszkowska and Majewska, 2014 and Samoliński et al. 2010 (Samoliński B, Rapiejko P, Lipiec A, Kurzawa R. Metody ograniczenia narażenia na alergen. In: Kruszewski J, Kowalski ML. Standardy w alergologii. Część I. Medycyna praktyczna, Kraków 2010.p.143–149 (in Polish).

9. **Materials and methods (lines 206-207): Is linear regression the unique method requiring parametric assumptions?**

Except for linear regression, the machine learning methods used in our study are nonparametric and do not require parametric assumptions. We have revised the manuscript accordingly to clarify this distinction.

10. **Materials and methods (line 267): I think "appropriate pollen concentration categories" is not the best statistical term.**

We have revised the term "appropriate pollen concentration categories" to "predefined pollen concentration classes (low, moderate, high)" in the manuscript. This change better reflects the categorical classification approach used in our predictive modeling.

11. **Results: Caption of Figure 5. "Betula" in italics.**

Updated the manuscript so that all text now appears in a consistent, standard font throughout.

12. **Results (lines 302-303): Why the specific results from the XGBoost are remarked and detailed, and not for the rest of the methods used?**

The XGBoost model was chosen to show the prediction example in Fig. 5 because it had the best prediction accuracy over this period. Showing examples for all models in a clear manner would take up a lot of space, so we decided to use one model.

13. **Results: Caption of Figure 6. "Betula" in italics.**

Updated the manuscript so that all text now appears in a consistent, standard font throughout.

14. **Results (lines 355-356): A reference is required.**

    Reference added.

We hope that the revised manuscript addresses all concerns raised by the reviewers and editor, and we believe it is now suitable for publication in *PLOS ONE*. We thank you again for your valuable feedback and look forward to your response.

Sincerely,

Daniel Bulanda

AGH University of Krakow
daniel@bulanda.net
