# Supplementary material for: Comparison of machine learning methods in forecasting and characterizing the birch and grass pollen season
Source: PLoS One. 2026 Feb 18;21(2):e0332093. doi: 10.1371/journal.pone.0332093 (PMC12915917; doi:10.1371/journal.pone.0332093)
Supplement: Attachment — Submitted filename: Response to Reviewers.docx [file pone.0332093.s004.docx]

**Response to Reviewers**

Manuscript title: *Comparison of machine learning methods in forecasting and characterizing the birch and grass pollen season*
Manuscript ID: PONE-D-25-12434R1
Journal: *PLOS ONE*

Dear Editor and Reviewers,

We appreciate your constructive feedback and are grateful for the opportunity to revise our manuscript. Thank you for your positive and valuable evaluation.

Below we address each point raised by the reviewers. All changes in the revised manuscript are highlighted in the “Revised Manuscript with Track Changes” file, and the clean version reflects these changes.

**Academic Editor Comments**

Thank you for your careful evaluation and for confirming that our manuscript is fundamentally suitable for publication pending minor revisions. We appreciate the insightful feedback from you and the reviewers. We will diligently address all comments and submit our revised manuscript by the deadline.

**Reviewer #1 Comments**

We are very grateful for your thorough re‐evaluation and kind assessment of our revisions. Your confirmation that we have coherently addressed concerns around measurement error and station-distance discrepancies, geographic generalizability, hyperparameter tuning, feature‐importance analysis, clinical implications, temporal synchronization, and consistency in meteorological factor interpretation is incredibly reassuring.

Your detailed recognition of our efforts to quantify microclimate differences, clarify methodological caveats, justify our modeling choices, and integrate secondary analyses means a great deal to us. Thank you for helping us strengthen the manuscript and for your constructive guidance throughout the review process.

**Reviewer #2 Comments**

We appreciate Reviewer #2’s encouraging feedback and thoughtful recommendations. Each of your points has been addressed in detail below. We are pleased that Figure 5 resonated with you. We’ve also implemented the minor clarifications listed to enhance clarity and precision.

1. **The authors restrict the application of the model to the pollen season, but the pollen season of birch and grasses was not defined. Have they have used any of the common methods to define the pollen seasons? It is relevant as the application of the method influences in the period selected (Tasioulis et al., 2022).**

Thank you for pointing out the need for greater clarity in our definition of the pollen season. We have rephrased the Materials and methods section accordingly to make explicit that:

- The birch and grass seasons begin on the first day with a pollen concentration > 0 grains/m³ and end on the last day with any detectable pollen, following Dahl et al. (2013).
- All zero‐count days that fall between these start and end dates were retained in the analysis.

Although our method does not employ any of the other widely used pollen‐season calculation algorithms, we elected to omit a detailed comparison of those approaches from the Materials and Methods.

2. **Based on the previous comment, check the clinical method due to the orientation of this manuscript in public health (Pfaar et al., 2017).**

Thank you for highlighting the importance of the clinical definition of pollen exposure. In response, we have added new sentences in the Discussion section summarizing the EAACI position paper by Pfaar et al. (2017), which recommends daily monitoring of pollen concentrations in immunotherapy trials.

We have added a statement noting that daily pollen monitoring, is crucial for accurately timing and assessing the effectiveness of specific immunotherapy for seasonal allergic rhinoconjunctivitis.

**Reviewer #2 Specific Comments**

1. **Abstract: "mean sea level", perhaps you mean "mean pressure at sea level".**
   Corrected.
2. **Abstract: "Ambrosia" genus in italics.**
   Corrected in the Introduction section, no such word in Abstract.
3. **Materials and methods: Replace "pollen counts" by "pollen concentrations".**
   Corrected.
4. **Results: "Betula" genus in italics.**
   All occurrences of the word *Betula* are in italics.
5. **Discussion: In which fields were MAGNs models used previously? Examples.**
   Multi-Associative Graph Networks (MAGNs) have been evaluated across a wide range of benchmarking tasks in the introductory publication (Horzyk A, Bulanda D, Starzyk JA. Construction and Training of Multi-Associative Graph Networks. In: Joint European Conference on Machine Learning and Knowledge Discovery in Databases. Springer; 2023. p. 277–292). The authors evaluated over 70 classification datasets from various fields, including medicine, technology, and biology, demonstrating MAGNs’ broad applicability across disciplines. Beyond benchmarking, MAGNs have also been applied in real-world, domain-specific projects. In the automotive industry, MAGNs have been used as recommendation engine and pattern mining framework at GrapeUp Ltd. In the medical domain, they are applied to the classification and analysis of electrocardiogram (ECG) signals in collaboration with Prof. M. Jastrzębski (Jagiellonian University) and Prof. J.A. Starzyk (Ohio University), with the goal of improving diagnostic accuracy and classification mechanisms. In the field of psychiatry, MAGNs are being implemented within the framework of the project MENTALIO – a decision-support system for diagnostics and therapy in adolescent mental health based on artificial intelligence algorithms, funded by the Polish Medical Research Agency (ABM/2022/7). The project is carried out in collaboration with Prof. M. Pilecki (Jagiellonian University) and the Nivalit company. It focuses on modeling complex psychological and behavioral data to predict children's and adolescents’ behaviors and to support suicide prevention. Notably, MENTALIO received the highest ranking in the national ABM competition for innovative AI-based medical technologies, with total funding of 3,768,206 PLN. These practical implementations further underscore the flexibility of MAGNs in handling diverse, high-dimensional, and interrelated data structures across a wide range of application areas.
6. **Discussion: Replace "grasses pollen" by "grass pollen".**
   Corrected.
7. **Conclusion: "mean sea level", perhaps you mean "mean pressure at sea level".**
   Corrected.
8. **Discussion: Replace "signWificance" by "significance".**
   Corrected.

We trust that these revisions address all the remaining concerns. Thank you again for your valuable feedback. We look forward to your decision.

Sincerely,

AGH University of Krakow
daniel@bulanda.net
