# Supplementary material for: Comparison of machine learning methods in forecasting and characterizing the birch and grass pollen season
Source: PLoS One. 2026 Feb 18;21(2):e0332093. doi: 10.1371/journal.pone.0332093 (PMC12915917; doi:10.1371/journal.pone.0332093)
Supplement: Attachment — Submitted filename: Response_to_Reviewers_auresp_3.docx [file pone.0332093.s005.docx]

**Response to Reviewers**

Manuscript title: *Comparison of machine learning methods in forecasting and characterizing the birch and grass pollen season*
Manuscript ID: PONE-D-25-12434R1
Journal: *PLOS ONE*

Dear Academic Editor,

We thank the Academic Editor for the thorough and constructive evaluation of our manuscript from the machine learning perspective. We appreciate the opportunity to improve the clarity, balance, and interpretability of our work. Below we respond point-by-point and describe all corresponding revisions implemented in the revised manuscript.

**Academic Editor Comments**

Below is a list of individual comments along with the authors' responses.

1. **There is an emphasis on the MAGN method, and more space is dedicated to explain it when compared with the other methods. I understand that it is a recently-developed method therefore most people won't know about it, but even so the difference on the details on it and the other methods is noteworthy. One of the authors of this paper is also an author of another MAGN paper used as reference to this one.**

We appreciate the Editor’s observation regarding the relative emphasis placed on the MAGN method. Our original intention in providing a more detailed description of MAGN was to ensure that readers unfamiliar with this recently developed approach could understand its structure and rationale, since unlike boosted trees or neural networks, it does not yet have widely available canonical references or textbook-style explanations. MAGN is conceptually different from the other methods evaluated and requires additional clarification to ensure transparency and reproducibility.

Importantly, our goal was not to privilege MAGN but rather to prevent ambiguity in the description of a method that most readers would be encountering for the first time. We also acknowledge that two of the co-authors previously contributed to the development of MAGN, which further motivated us to provide an especially clear and self-contained explanation so that the method could be independently assessed.

To address the concern regarding imbalance, in the revised manuscript we have added one additional paragraph to both the linear regression and k-NN method descriptions to provide clearer methodological balance, as these two models were previously underrepresented relative to the others. This ensures that all methods are presented with a more comparable level of detail while still providing the clarity necessary for readers to understand and replicate the MAGN approach.

2. **Figure 2, that describes MAGN, is not really useful, more details are needed. The text that refers to the figure is also not so clear. MAGN shows best or second-best results in mean accuracy, mean MAE, mean execution time and mean memory consumption. From the results it seems very impressive, but the lack of details on the method may hinder its adoption by other researchers.**

We thank the reviewer for pointing out that Figure 2 and its accompanying description lacked sufficient detail. In the revised manuscript, we have substantially improved the caption of Figure 2 to clearly explain the roles of sensory fields, sensory neurons, object neurons, duplicate counters, defining connections, and similarity connections within the MAGN architecture. In addition, we have added a new explanatory paragraph in the text that walks the reader through the structure step-by-step and clarifies how these components interact during learning and inference. These revisions enhance the clarity and interpretability of the figure and provide a more accessible explanation for researchers who may not yet be familiar with MAGN.

3. **Table 3 shows the source code file name for each method, with references to the source code (e.g. 35, 36, 38) that are not really useful. Why not use a reference to the canonical paper or book that describes the method? Links to the source code could be provided as additional references or footnotes.**

We thank the reviewer for this helpful suggestion. In the revised manuscript, we have added canonical theoretical references for each machine learning method listed in Table 3, ensuring that readers can easily locate the foundational literature describing each model. We have also included a link to the MAGN implementation, consistent with the other methods, and extended the table caption to clarify the purpose of the theoretical references and implementation links. These changes improve both the scientific rigor and the reproducibility of the presented methods.

4. **Explanation for figure 7 could be more clear: not only comment on the graphical representation of the plot but also point to points of interest on it. In other words, which conclusions are supported by that chart's features?**

We thank the reviewer for noting that the interpretation of Figure 7 required greater clarity. In the revised manuscript, we have expanded the description of Figure 7 to go beyond the graphical explanation and now explicitly highlight the most important patterns visible in the plot. We identify which meteorological combinations lead to high-confidence and high-lift rules, describe the differences between *Betula* and Poaceae, and explain how the size, position, and color of the points support our conclusions. This updated interpretation makes the chart more informative and demonstrates how the association rules contribute to understanding the meteorological drivers of pollen concentration.

5. **Figure 6 shows that pollen concentration is the main predictor for pollen concentration... this happens for seasonal predictions. The most important applications are related to prediction of when something will start and stop, which I expected to be the case for this paper (see introduction, line 8). What would happen if we didn't use pollen concentration as an input variable?.**

We appreciate the reviewer’s insightful comment regarding the use of past pollen concentrations as predictors and the relevance of forecasting the beginning and end of the pollen season. We agree that onset and offset prediction is an important application. However, the scope of the present study is limited to within-season forecasting and early-season characterization, as stated in line 8 of the introduction. The phrase “early characterization of the upcoming pollen seasons” refers specifically to modeling pollen behaviour after the first measurable occurrence, not to predicting the initial onset itself.

For this reason, experiments excluding pollen concentration as an input variable, while highly valuable for onset/offset prediction, fall outside the objectives of this work. Nevertheless, we acknowledge the importance of the reviewer’s suggestion. Our team is currently developing a follow-up study focused explicitly on pollen season start and end prediction and on evaluating models without historical pollen inputs. We expect this new study to be completed within this year and believe it will address the reviewer’s points in full.

To improve clarity, we revised the sentence to explicitly state that our work focuses on the prediction and characterization of pollen levels after the season has begun. This modification aligns the introduction with the scope of the study and prevents misunderstanding regarding onset prediction.

6. **About the title: "Comparison of machine learning methods in forecasting and characterizing the birch and grass pollen season" -- I didn't see much of "characterizing of the seasons", as I expected from Experiment 2, which deals with "characterize the relationships between the input and target variables" but without a good, interpretable explanation -- just a description on lines 360-362, which seems to leave the task to the reader.
   Overall, considering the ML methods and applications, I think clearer explanations would help. The problem the authors want to solve is clear (predict pollen concentration), the variables are well explained, but the paper title ("Comparison of machine learning methods in forecasting and characterizing the birch and grass pollen season") seems too broad and results could be better explained.**

We appreciate the reviewer’s observation regarding the “characterizing” component referenced in the title. In the revised manuscript, we have expanded the interpretation of Experiment 2 to provide a clearer and more explicit characterization of the relationships between meteorological variables and pollen concentration classes. Additional text now highlights the most informative association rules, explains how these rules reveal underlying seasonal patterns, and connects these findings to established aerobiological knowledge. These enhancements ensure that the characterization aspect of the study is more transparent and accessible to the reader.

We acknowledge that the original presentation placed more emphasis on forecasting results, which may have contributed to the perception that the title was broader than the content. With the strengthened explanations, the paper now more fully reflects both elements, forecasting and characterizing, as indicated in the title.

7. **Access to the whole dataset is somehow restricted, rules and contacts to getting to it are listed in the documents (but not on the paper?), I sort of expected a better explanation on why the full data is not available, but I also think this is not an issue that would hinder the publication of the work since there are links to a smaller version of the dataset that allows for replicability.**

We thank the reviewer for raising this point. The long-term pollen monitoring data are owned and curated by the Department of Clinical and Environmental Allergology at the Jagiellonian University Medical College, and due to institutional data-governance policies and confidentiality considerations, cannot be made openly available in their entirety. Access requires a formal request, a brief research plan, and confirmation of compliance with data-use and confidentiality rules. To the best of our knowledge, the Data Availability section is the proper and expected location for providing these explanations.

To ensure reproducibility despite these restrictions, we have provided a publicly accessible minimal dataset (including 2022-2023 data points). This subset is sufficient to replicate all analyses and modeling procedures presented in the paper.

8. **There are still minor issues with the format of the text, notably in tables.**

We thank the reviewer for noting the remaining formatting issues. In the revised manuscript, we have reviewed and corrected all identified formatting problems in the tables and surrounding text.

We hope these revisions resolve the remaining issues. Thank you for your thoughtful feedback. We look forward to your decision.

Sincerely,

AGH University of Krakow
daniel@bulanda.net
